# Two kinds of evolutionary individuals: the concept of common interest as an evolutionary foundation of dualism on biological individuality

**DOI:** 10.1007/s40656-026-00721-w

**Published:** 2026-02-24

**Authors:** Adrian Stencel

**Affiliations:** Institute of Philosophy, Jagiellonian University, Kraków, Poland

**Keywords:** Organism, Immunology, Ecology, Development

## Abstract

What is a biological individual? This is a question that has been of interest to biologists and philosophers for a long time. The usual response is an attempt either to find a single, unifying concept (a monistic stance) or to justify the existence of multiple concepts, for instance, by referring to scientific practice (a pluralistic stance). In this paper, I adopt a pluralistic stance and focus on evolutionary studies. I argue that in the context of evolutionary biology we need to distinguish between two kinds of evolutionary individuals, based on the concept of common interest. I decouple this into common interest by necessity (CIN) and common interest by contingency (CIC), and argue that this division corresponds to two kinds of evolutionary individuals. To situate the framework I thus develop within the context of ongoing discussions, I then compare it with frameworks corresponding to other concepts commonly found in the relevant literature, such as those concerning units of selection and agency. The conclusion is that the ontology of evolutionary biology is more complex than often assumed, and that we should distinguish: units of selection, agents, and two kinds of evolutionary individuals.

## Introduction

When we observe a single ant or bee, it looks like an individual as it moves, responds to stimuli and interacts with others; but when we contemplate the whole ant or bee colony, that somehow resembles an individual as well (Boomsma & Gawne, 2018; Helanterä, 2016; Seeley, 1989). The same problem can emerge when we shift our attention to other biological entities: symbiotic associations (Skillings, 2016; Suárez, 2020), fungi (Booth, 2014; Molter, 2020), even plants (Clarke, 2012; Gerber, 2018), etc. So, we may well ask: What is a biological individual? This question is interesting for philosophers, because it concerns understanding the very nature of the biological units that populate our world (Bouchard, 2014; Dupré, 2010; Fábregas Tejeda et al., 2023; Godfrey-Smith, 2013; Wolfe, 2014). It is also of great importance for scientists, because the concept of individual plays a role in many biological debates (Bordenstein & Theis, 2015; Helanterä, 2016; Pepper & Herron, 2008). For instance, if we want to evaluate population size, as in many research studies (Lanfear et al., 2014; McCarthy & Thompson, 2001), we must assume or in some way define what a biological individual is. In evolutionary terms, understanding which individual is fitter (Hammerschmidt et al., 2014; Matthen & Ariew, 2002), can only be addressed if we know what this individual is, to which we assign degrees of fitness. The same goes for physiological studies: certain aspects of animal physiology only became clear when scientists switched to the idea that an individual animal consists of a host and symbiotic microbes (Bordenstein & Theis, 2015; Brucker & Bordenstein, 2013).

The previous paragraph shows that understanding the notion of a biological individual is an interesting and important issue. The issue has been puzzling scholars for a long time and many attempts to solve the problem have been developed (e.g., Dupré, 2010; Godfrey-Smith, 2009; Hull, 1980; Moreno & Mossio, 2015; Pradeu, 2019). We can divide these into two main approaches. On one hand, some people tried to find the definitional concept that would play a universal role (e.g., Clarke, 2013; Gardner & Grafen, 2009). For instance, Clarke argued for just such a monistic stance, stating that evolutionary biology could provide us with the required concept. On the other hand, certain scholars were more interested in justifying having multiple concepts of biological individuality (Kaiser & Trappes, 2021; Kovaka, 2015; Pepper & Herron, 2008; Pradeu, 2016b; Suárez & Lloyd, 2023). For instance, Kovaka (2015) argued for such a pluralistic stance and justified it by reference to scientific practice: the biological sciences need many concepts because they assist scientists in achieving different goals.

Interestingly, when issues related to biological individuality are discussed, scholars very often combine two types of entities to build a framework for their research purposes. For instance, Godfrey-Smith (2013) distinguishes between Darwinian individuals and organisms. Smith (2017), building upon Godfrey-Smith’s framework, then distinguishes between Darwinian individuals and “persisters”; the latter being her specific elaboration of Godfrey-Smith’s concept of organism. Stencel and Wloch-Salamon (2018) made a distinction between Darwinian individuals and “organismality”, as initially developed by Queller and Strassmann (2009). O’Malley (2016) lists multigenerational and multi-lineal reproducers; while Dupré and O’Malley (2009) make a distinction between lineage-forming units and metabolic wholes. Pradeu (2016a, 2016b) makes a compelling case for physiological and evolutionary individuality, while Stencel and Proszewska (2018) provide arguments in favor of distinguishing between developmental individuals and, again, the concept of organismality. This trend suggests that for research or philosophical analysis, it is usually sufficient for scholars to rely on just two concepts of biological individuality. This, in turn, raises further philosophical questions. For instance, do these frameworks represent genuine dualistic stances, and as such should they be placed in opposition to the monism and pluralism that I outlined above? Or do they belong to the pluralistic paradigm? There is probably no simple answer. Pradeu (2016a, 2016b) openly recognizes his pluralistic commitments, while Godfrey-Smith (2013) avoids any such stance. Therefore, it is hard to make strong generalizations, beside the fact that relying on two concepts of biological individuality seems to be recurrent.

In this paper, I assume that dualistic frameworks can be understood as part of a pluralistic stance, where instead of picking a single concept from the many available, scientists pick two and combine them, as this is useful for their research. Given this, I will focus on a different issue, but one related to these dualistic frameworks. I think that contemporary approaches generally suffer from a certain issue: they combine concepts from different fields to build their dualistic frameworks (see Godfrey-Smith, 2009; Pradeu, 2016a; Stencel & Proszewska, 2018). While I think this can be an interesting approach that might be useful for interdisciplinary studies, I believe its utility is limited when it comes to more specific studies. The reason for this stems from the fact that such approaches combine concepts that were developed to be used for achieving different scientific goals: to study immunology (e.g. Pradeu, 2010), development (e.g. Gilbert et al., 2012) or evolution (e.g. Godfrey-Smith, 2009), for instance. Therefore, applying them to a single research goal might be problematic. In cases where work within a given field requires the use of more than one concept, I think it would be better to select concepts based on the same principles, thereby maintaining the cohesion of the research and the integrity of the results.

So, I aim to develop a dualistic framework for evolutionary biology, for three main reasons. Firstly, as mentioned above, dualistic frameworks are common; so, further analysis of their nature would enhance our understanding of their role and place in biology. Secondly, such a framework could provide a cohesive evolutionary worldview. Currently, dualistic frameworks mix evolutionary concepts of individuality with non-evolutionary concepts (Godfrey-Smith, 2013; Okasha, 2023; Pradeu, 2016a), which might decrease their operability for scholars. For instance, evolutionary biology is interested in analyzing fitness (Hammerschmidt et al., 2014; Jeler, 2024; Matthen & Ariew, 2002), which could be done using evolutionary concepts of individuality since fitness plays an important role in these concepts (Bourrat & Griffiths, 2018; Godfrey-Smith, 2009; Okasha, 2006). In contrast, assigning fitness to non-evolutionary concepts of individuality is highly problematic as those concepts were designed to capture different phenomena and reference to fitness plays no theoretical role in them (see Pradeu, 2010; Suárez & Stencel, 2020). Therefore, there is a need for a dualistic framework that could be used for a single research goal within the field of evolutionary biology for the same research goal. Thirdly, many evolutionary models already assume two types of biological entities. Tarnita et al. (2013) distinguish between “stay together” (ST) and “come together” (CT) units, while Heisler and Damuth (1987) differentiate “multilevel selection 1” from “multilevel selection 2.” A dualistic framework in evolutionary biology could offer a metaphysical basis for such models and potentially lead to new insights.

How should we proceed with building evolutionary dualism on biological individuality? One promising idea that might assist us here is the concept of ‘common interest’. Reference to common interest is widespread in evolutionary biology and beyond, especially when scholars talk about individuality in the context of evolutionary theory (Leigh, 1991; Szathmary, 1991; West et al., 2015), the concept of agency (Aanen et al., 2023; Okasha, 2018) or sometimes during the debate concerning the units of selection (Foster et al., 2017; Skillings, 2016; Stencel & Wloch-Salamon, 2018). The same concept also appears in the context of the study of genetic conflicts within an organism (Leigh, 1977; Scott & West, 2019); interactions between mother and fetus (Haig, 1996; Roberts et al., 1996); and mutualistic interactions between different species (Leigh, 1991; Leimar & Hammerstein, 2010). It crops up outside biology as well, for instance, in economics (Fennell, 2003) and sociology (Stark & Flache, 2012). There certainly seems to be something quite unique about the concept.

The aim of this paper is therefore to build a dualistic stance on biological individuality based on evolutionary principles. To do this, the paper is structured in the following way. I will rely on the concept of common interest; so first, I present this in the next section. Then I decouple this concept into common interest by necessity (CIN) and common interest by contingency (CIC), which I will argue capture two different kinds of biological individuality present in the evolutionary realm. Finally, I will review some of the differences and similarities between my dualistic framework and some others that are present in the literature, and I will discuss some related consequences.

## The concept of common interest

Before we consider how best to think about ‘common interest’ in the context of evolution, it is interesting to see how people who are not working in evolutionary biology understand this concept. It is usually used to emphasize the fact that certain groups share a common goal: there is something that all group members want to achieve. As a result, typically, members tend to cooperate and resolve potential conflicts in order to reach the common goal. Political machinations in democratic states are one place where this is easy to spot. As different groups will benefit from some specific legislation, they share a common interest in having that legislation in force (e.g., Bawn, 1999; Noel, 2012). As a result, those groups will tend to support each other with regard to the legislation in question, even though they might not agree in other areas. This raises a question: What common goal might entities share in the context of evolution?

It seems clear that the most appropriate way to look for an answer to this question is to focus on the concept of natural selection, which is the basis of the theory of evolution, and ask: What are the fundamental conditions for natural selection to occur? There has been a tremendous amount of effort spent analyzing natural selection in many aspects, from mathematical models (e.g., Frank, 2003; Tarnita et al., 2013) to empirical experiments (e.g., Hammerschmidt et al., 2014; Ratcliff et al., 2012) and philosophical investigations (e.g., Bourrat, 2014; Okasha, 2006). Regardless of the many disagreements in evolutionary biology, scientists tend to agree that at the heart of natural selection lie differences in fitness, i.e., variation in rates of survival and reproduction. References to this can be found in all fields of discussion concerning natural selection (e.g., Doolittle & Booth, 2017; Futuyma & Kirkpatrick, 2017; Godfrey-Smith, 2009; Lewontin, 1970; Okasha, 2006; Orr, 2009). Such variation seems to be crucial for natural selection to take place, because differences in rates of survival or reproduction will make certain types of entities become more frequent in the population over time.

Of course, how a population evolves depends on many circumstances (see Godfrey-Smith, 2009); but we can clearly state that without differences in fitness, there will be no natural selection. As Orr (2009) stated: “Without differences in fitness, natural selection cannot act and adaptation cannot occur.” Note, however, that this will not only depend on differences in direct fitness (i.e., surviving and reproducing), but also on indirect fitness (i.e., nurturing offspring). The combination of these two aspects has been baptized “inclusive fitness” (Hamilton, 1964), and this concept has helped to explain the evolution of such complex biological phenomena as altruism in eusocial insects (Abbot et al., 2011; Gardner & West, 2014). In light of this, I think it is justified to say that differences in inclusive fitness form the foundation of natural selection.

At this point, I believe we can agree that: (1) common interest is like a shared goal, something that the parties want to achieve via partnership, and (2) scientists tend to agree that natural selection occurs when some types of entities have greater inclusive fitness. This suggests that if there is any goal that an entity could *want* to reach from an evolutionary perspective, it must be one that increases its inclusive fitness, because those that reach this goal will become more common in the population over time. Therefore, I think it is fair to say that from an evolutionary perspective, a given aggregation of entities share a common interest if their inclusive fitness is mutually linked, i.e., when an increase/decrease in fitness for one entity implies a change in the same direction for the rest. Of course, the magnitude of the change varies and depends on the nature of the system; but I think what matters is that the move is in the same direction because this suggests that the entities are connected in a way that matters from an evolutionary perspective (see Bourrat & Griffiths, 2018).

The way I framed common interest above is not in any way unique, but rather it follows a long tradition of thinking about evolutionary units in the context of evolutionary biology. Similar ideas have been expressed before, in one or another way, by many scholars. Some, as I do, call it common interest (Leigh, 1977, 1991; Leimar & Hammerstein, 2010; Skillings, 2016; Stencel & Wloch-Salamon, 2018), others use different names such as “fitness alignment” (Bourrat & Griffiths, 2018; Douglas & Werren, 2016; O’Malley, 2016; Van Baalen, 2013), but all refer to the idea that some sort of strong linkage, that matters from an evolutionary perspective if we are to consider something a meaningful unit, is concerned with fitness. For instance, Leimar and Hammerstein (2010, p. 2620) state: “Common interest means, in its most extreme form, that reproductive success is fully linked among the interactants, so they stand or fall together”. However, I think that this way of thinking is incomplete, because it does not take into account the idea that a common interest might be shared by entities that are parts of very different types of systems. To fill this gap and elaborate on the concept of common interest, I want to draw a distinction between two kinds of common interest, as I advanced above: common interest by necessity (CIN) and common interest by contingency (CIC). I think these two kinds of common interest describe systems that are so different that it is justified to assume that we should consider them as capturing two different kinds of biological individuals. Here, a system is understood in a minimal sense, i.e., as a set of interacting entities (see von Bertalanffy, 1968).^1^

As I move forward with this analysis, this seems to be an appropriate point to state what kind of strategy I have decided to adopt in order to decouple the concept of common interest into two kinds. I will focus on the autonomy of the parts of the system. There is a broad discussion on autonomy in biology, concerning many different aspects (Fulda, 2023; Moreno & Mossio, 2015). Here I understand the autonomy of a part as being in possession of the biological mechanisms that allow it to leave the system and live outside, on its own, or the capability to be integrated into another system. I think once a system is established there are, ontologically speaking, just two paths that the system can take in relation to the autonomy of its parts. These options represent two different kinds of biological individuals, from the evolutionary perspective. In the next section, I will start by presenting the more restrictive option and then move on to the more relaxed one.

## Decoupling the concept of common interest

### Common interest by necessity

**Fig. 1:**
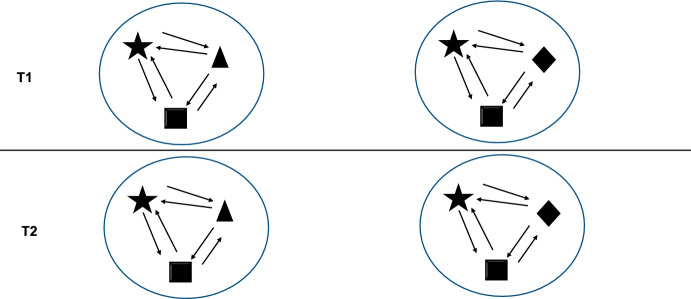
Common interest by necessity (CIN) refers to the system where an increase/decrease in inclusive fitness of some parts (represented by two-way arrows), implies the same direction of change in inclusive fitness for other parts of the system. The autonomy of parts is constrained; once the system is established, there are no mechanisms that allow parts to leave and integrate into another system (represented by circles with solid lines). As a result, there is no movement of parts from one point in time to another (T1 and T2)

A fundamental question is: Why do some entities share a common interest? I think one reason this happens is due to the evolution of complex developmental cycles (e.g. Griesemer, 2014; Hall, 2012). Many biological systems do not initially appear in the form in which we observe them. Complex multicellular animals are paradigm examples of this (Hall, 2012; Swartz & Wessel, 2015): they pass through different stages of development. During that development, certain parts emerge; they are assigned functions and start to perform a specific role within a given location of the system. To perform these functions, the parts must be highly specialized, and over the course of evolution they lose the ability to withdraw from the system (Fig. 1). Consequently, evolution has produced complex entities composed of specialized parts that arise during developmental processes and cannot detach from the system to which they belong. Furthermore, many biological systems have evolved numerous barriers that separate them from similar systems, so that when two such systems meet, there is very little exchange of elements between them; and even if this does happen, the chance of integration is very small. In such a case, the different parts of the system are highly restricted. There are no mechanisms that allow them to withdraw from the system and lead a separate life or become integrated into another similar system.

In this case, the only way the parts that make up the whole can survive and reproduce, is if the whole succeeds. Such systems realize common interest by necessity (CIN) and can be summarized as follows:

1. When a group of entities constitutes a system, an increase/decrease in inclusive fitness of some parts, implies a change in inclusive fitness in the same direction for all other parts of the system.
2. The autonomy of the parts is constrained: once the system is established, there are no mechanisms that allow parts to leave and live on their own or to become integrated into another system.

Necessity is not understood here in a deep metaphysical sense (e.g. Cameron, 2010; Ditter, 2022). It simply means that as some entities start to interact and form a sort of system comprised of elements that share a common interest, they lose the capacity to leave that system. The reason for this is that there are no biological mechanisms that would allow for such a transition, which would be followed by smooth integration into a similar system. Furthermore, due to the complex development that brought them into existence and transformed them into sophisticated parts, they are unable to start a single, unattached life. As a result, they are doomed to thrive or fail with the system. Thus, their common interest is by necessity.

The paradigm example of CIN would be complex multicellular animals such as cats, cows or humans made of multiple cells that perform a variety of functions within the whole. Some cells are responsible for digestion, some protect the whole from pathogens and others perform functions that allow movement from one place to another. The thing about such a system is that there are structural constraints on its constitutive elements that impede them from leaving the system. During the development of such a complex animal, cells are assigned to perform certain functions; some cells develop into immunological cells, and some into neurological cells, etc. (Hall, 2012). This division of labor is beneficial for the whole, as it leads to a specialization of elements into certain functional units (like the immunological system, brain, etc.); at the same time, this specialization of cells makes it impossible for them to leave the system. For example, a neurological cell cannot just leave the body of a cat and initiate a free-living life, as bacterial cells can. It is even impossible for a cell to leave one system and join another: barriers such as skin or the immunological system effectively prevent cells from leaving one such system and entering another.

The cells that belong to a cat's body cannot leave it and join another cat's body. They do not have the requisite biological capabilities. Therefore, for the cells of the cat to increase their fitness, they have to interact with each other: the structural constraints prohibit them from withdrawing from the system. Thus, the only way they can succeed is if the whole succeeds. This is why complex multicellular animals are paradigm examples of CIN. However, increasing their fitness does not happen through a direct increase in their survival or reproduction rate, as only a fraction of the cells reproduce directly to form a descendant cat: germline cells. Rather, it is assumed among scientists that all cells benefit indirectly, as they are genetically very similar, and so by helping relatives (i.e., somatic cells contributing to the success of germline cells) they increase their inclusive fitness because they help to spread their genes through indirect means: helping their relatives that carry those same genes. It should be noted here that this is considered the main reason why multicellular organisms are genetically homogeneous (Fisher et al., 2013; Maynard-Smith & Szathmary, 1995; West et al., 2015), but not the only one (Ågren et al., 2019; Patten et al., 2023; Stencel & Suárez, 2021; Suárez et al., 2025). This entails that the cells of a cat, for example, clearly share a strong common interest: if the inclusive fitness of muscle cells is increased/decreased, then it follows that other cells will experience a similar gain/loss, as it means that the cat is more/less likely to successfully reproduce.

### Common interest by contingency

**Fig. 2:**
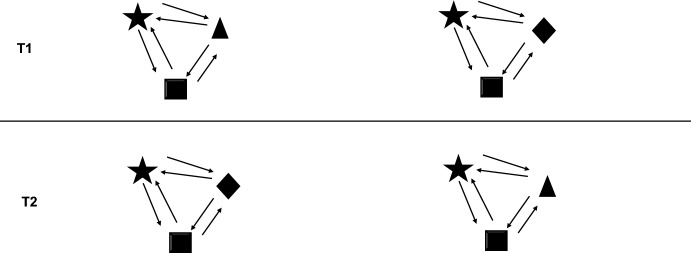
Common interest by contingency (CIC) refers to the system where an increase/decrease in inclusive fitness of some parts (represented by two-way arrows), implies the same direction of change in inclusive fitness for other parts of the system. The autonomy of the parts is not constrained; once the system is established, there are mechanisms that allow parts to leave and integrate into another system (represented by interacting elements that are not in a circle). As a result, there is movement of parts from one point in the time to another (T1 and T2)

The kind of common interest explained in the previous subsection is not the only one that we encounter in nature. Natural selection also leads to the evolution of systems that are more flexible and loose in terms of their integration. In some cases, the inclusive fitness of entities might be strongly linked when a system is established, however, there are no general biological constraints that preclude the possibility of abandoning a system and establishing a new one (Fig. 2). This can happen when biological associations come into existence through aggregation, which might lead to the formation of important evolutionary and biologically groups that are, nonetheless, very loose. It is possible that some parts of a given system could move between different systems and easily become integrated within them. This is why such systems are examples of CIC. In this kind of systems, the inclusive fitness of the parts is linked (sometimes even tightly), but they form part of the same system due to contingent events: due to their particular life history, which could have changed and still can change in the future, as the parts retain the ability to withdraw from their current association. This contrasts with CIN, where the parts are constrained within a given system, because in CIC certain biological characteristics permit a transition to a different system.

Consider a nerve cell in a cat. Even if certain circumstances had been different during the cat’s life, its nerve cell could not have been part of the nervous system of a different cat. In contrast, in the case of systems characterized by CIC, such things are possible and so they can be summarized as follows:

1. When a group of entities constitutes a system, an increase/decrease in inclusive fitness of some parts, implies a change in inclusive fitness in the same direction for all other parts of the system.
2. The autonomy of some parts is not constrained: once the system is established, there are mechanisms that allow parts to leave and live on their own or to become integrated into another system.

Paradigm examples of systems that exhibit common interest by contingency include many kinds of symbiotic interactions (but not all) between multicellular hosts and their microbial partners acquired from the environment during the host’s life (e.g., Amato et al., 2021; Bordenstein & Theis, 2015). Animals lacking specific microbes suffer from many physiological deficiencies, which influence their fitness, as documented by studies that compare wild animals to their laboratory counterparts with modified microbiomes (see Rosshart et al., 2017). A good case that exemplifies the importance of symbiotic microbes for the fitness of multicellular organisms is that of the interactions between mice and their symbiotic bacteria, *Bacteroides fragilis.* These bacteria are located in the gastrointestinal tract of mice and produce polysaccharide A (PSA). PSA directs the cellular and physical maturation of the developing immune system, which includes correction of systemic T-cell deficiencies and Th1/Th2 imbalances in lymphoid tissues, which are typical in germ-free mice (Mazmanian et al., 2008). Additionally, *B. fragilis* contributes to providing the host with food, as it participates in the process of digesting nutrients and potentially plays many more roles in the physiology of the host (Wexler, 2007; Zafar & Saier Jr, 2021). Therefore, the presence of *B. fragilis* makes a positive contribution to the inclusive fitness of mice because the immunological system is of crucial importance for their survival and reproduction.

*Bacteroides fragilis* also benefits, in terms of the inclusive system, from being part of such a system, because it is adapted to living in the environment that the mouse gut provides: it is an anaerobe microbe that is unable to reproduce outside the host (Smith et al., 2006; Wexler, 2014). However, it can survive outside for some time, as Rocha et al., (2003, p. 167) put it: “*B. fragillis* is one of the most aerotolerant anaerobes being able to survive (but not multiply) for 48–72 h in the presence of atmospheric O2”. In this context, it is not controversial to say the two share a common interest, because a change in the fitness of one will imply a change in the same direction in the fitness of the other. For instance, if a predator kills the host and decreases its inclusive fitness, this will imply a change in the same direction in the inclusive fitness of the bacteria, because they are unable to reproduce outside the host. Alternatively, if germ-free mice pick up *B. fragilis*, this will increase their inclusive fitness due to improving immune system functionality and the fitness of *B. fragilis* will increase as well, because it can only reproduce inside the host.

The preceding description marks the fundamental difference between CIN and CIC. The former concerns systems constituted of parts that share a common interest, but once those systems are established constraints on their parts prevent them from leaving the system. In the latter case, we can also have systems of entities that share a common interest when they establish interactions; yet, they are constituted of parts which have more autonomy. In general, in this latter case, certain biological mechanisms allow for the movement of parts between such systems or even allow them to leave the system and live on their own, given the appropriate conditions. Let me return to the previous example to illustrate this. First of all, *B. fragilis* occupies the gastrointestinal tract of different species, such as mice (Rosshart et al., 2017), humans (Mazmanian et al., 2008), bears (Sommer et al., 2016) and pigs (Tajima & Aminov, 2015). Moreover, different species of microbes could play the same role in the physiology of the host (Suárez, 2020; Taxis et al., 2015). Secondly, these microbes can be transmitted within populations via contact between individuals like, for instance, via food sharing (Sarkar et al., 2020; Tung et al., 2015). Given these two facts, we can imagine a world where *B. fragilis,* which is present in the gut of certain mice, could move to another host, thanks to contact between hosts, and continue to perform its important function there, improving the new host’s fitness. In such a case, *B. fragilis* would also share a common interest with the new host, because the bacterial species in question is unable to lead a complete life outside its host. Yet, as illustrated, they are not inseparable. Even though they share a common interest when they are part of the same system, this common interest has a contingent character: if certain circumstances occur, the bacteria can switch between systems or the CIC can even be broken down under certain circumstances.^2^ Indeed, the bacteria have biological properties that allow them to leave the system and establish a new one with different partners. So even if the host dies, or the microbes die out, the remaining parts could still rebuild the system and incorporate new parts from outside. This is in sharp contrast with CIN, which binds parts to the extent that they are irreplaceable. For example, if you remove all neural cells from a mature cat (or the majority of them), they could not be replaced and this would lead to the collapse of the system. This all goes to show that CIN and CIC represent, ontologically speaking, two different kinds of common interest in nature. Therefore, I think it makes sense to consider the systems exhibiting them as two different kinds of biological individuals.

### The dynamic nature of common interest 

The way I have presented the two kinds of common interest might suggest that they are fixed: once a given system is identified as representing CIN or CIC, it cannot be changed. Indeed, apparently it should be that way, because whether something is classified as CIN or CIC depends on whether its parts have some sort of autonomy or are constrained by the system they form part of. The distinction thus relies on the existence of certain biological mechanisms and a certain type of fitness linkage. However, we should be aware that biological mechanisms and fitness linkage are things that change over time: they might emerge or vanish. Therefore, whether a given system exhibits CIN or CIC is usually true for a certain period of time. Sometimes, this characteristic is stable on the evolutionary scale; sometimes, the system might break down within a single generation.

The evolution of multicellularity is sometimes referred to as one of the major transitions in evolution, because it transformed free-living cells into complex higher-level wholes (e.g. Maynard-Smith & Szathmary, 1995; Michod, 1999). Normally, once the step has been taken, it is irreversible; this is the case of cat cells, which are unable to revert to single-cell life. However, in very rare cases, cells can regain some autonomy. This is the case of a devil facial tumor (DFT): a transmissible cancer that occurs in Tasmanian devils (Stammnitz et al., 2023; Taylor et al., 2017). The cancer cells can move between Tasmanian devils through intimate contact, such as biting. This means that they can move from one Tasmanian devil to another and multiply there, damaging the body they reside in. As a result, their inclusive fitness is no longer linked with that of the rest of the cells in the body they occupy at that moment: the common interest is broken. They are still dependent on the host; however, the fitness of these cancer cells and that of the Tasmanian devil is not directionally linked, as the cancer cells impact negatively on the host fitness, so they do not share a common interest anymore. This case is similar to that of many parasites that are dependent on their host, but the host does not benefit from them (Choe et al., 2019; Dowell & Ho, 2004; Stencel, 2021). In such cases we should not talk about common interest. Another example concerns gaining the status of CIN. Many symbiotic microbes have a loose interaction with their host: they can enter the body and then leave, so they might share CIC to some extent. Sometimes this can change, though; this is the case, for instance, of the integrated symbiotic bacteria in aphids, *Buchnera sp.*, which are now transmitted across generations in specific propagules called bacteriocytes. Furthermore, both partners remain in strict metabolic interaction, as the host provides defense and nutrients, and the bacteria synthesize amino acids that the host diet lacks. As a result, the fitness of the partners is directionally linked (see, Bennett & Moran, 2015; Chong et al., 2019). Furthermore, neither host nor bacteria can swap partners due to the high level of integration. Therefore, they now represent an example of CIN.

The previous paragraph describes a dynamic change that might occur in some systems and which happens over an evolutionary time scale. However, similar changes can take place on a shorter time scale. This might be the case for many systems that exhibit CIC. In such systems, we expect that when they are established, parts can still withdraw from the system and become integrated into another, or even live on their own, given appropriate conditions. Therefore, associations of this kind might be of just a temporary nature. This opens up the possibility of breaking down fitness directional linkage as well: excessive multiplication of one entity might have a negative impact on the other, which might still provide some benefit to the first one, if appropriate circumstances occur. For instance, the presence of *B. fragilis* in the gastrointestinal tract of different species (e.g. Mazmanian et al., 2008; Rosshart et al., 2017; Sommer et al., 2016), as mentioned in the previous subsection, is beneficial for the partners and leads to directional fitness linkage. Yet, *B. fragilis* can leave the gastrointestinal tract and move to another part of the body if intestinal integrity is damaged. Such damage can arise from rupture of an inflamed appendix, physical injuries, colon cancer or surgery (see Patrick, 2022). This could have a negative impact on the host, as it might lead to serious pathologies, including bacteremia and abscess formation at multiple body sites (Patrick, 2022; Wexler, 2007). As this might even be lethal and lead to a decrease in the host’s inclusive fitness, this is usually not beneficial for *B. fragilis,* since it can only survive outside a host for up to 72 h (Rocha et al., 2003). Indeed, if the bacteria decrease the fitness of their host to zero, the same decrease in their fitness might follow. This is the case unless there is a high chance of being picked up by a different host, which is possible via a movement of microbiota among a population (Sarkar et al., 2020; Tung et al., 2015). In such a scenario, damaging the host still might be beneficial for the bacteria, if this allows them to leave the host and colonize many other hosts sufficiently fast, which could occur if the population density is sufficiently high. This is, of course, only a hypothetical example, but it shows that when we handle systems that exhibit CIC, the common interest might be broken if suitable conditions emerge. By their very nature, such systems are just looser; in contrast to the case of CIN, CIC characterizes systems that might be of a temporary nature.

This section shows that CIN and CIC can demonstrate dynamism, both on a short and a long time scale. This is what we should expect from the biological world. The strength of the fitness linkage, its stability or the ability to withdraw from a system vary in different biological cases. This is because biological phenomena move across the landscape of possibilities, from one point to another, with many intermediate cases. This suggests that there will be many cases that are somehow in the middle, where it is very difficult to assign a given system either to the CIN or CIC category. For example, multicellular plants with their modularity that significantly differ from complex multicellular organisms (Clarke, 2012; Gerber, 2018; Yilmaz & Dupré, 2025) might pose such a problem. As Ellen Clarke (2011, p. 6) put it: “An oak tree is a unitary organism to a large extent. (…) Cells taken from just about any part of the tree can, given the right conditions, be grown into a whole new and sexually fertile tree. You cannot do this with most metazoans.” Such systems tend to be more flexible and so their parts are more autonomous. However, not all their parts are as free to move as the bacteria that live on human skin, which can move from one host to another easily. Due to the developmental processes, certain parts of some species might lose the ability to leave the plant and start a new one, such as leaves in nonsucculent species (see Gorelick, 2015). Therefore, many multicellular plants would be located somewhere between the paradigmatic CIN and CIC cases that I discussed in the last section. However, this does not cast doubt on the evolutionary dualism I advocate in this paper. There are still two fundamental kinds of common interest, it is just that the gray zone between them might be very colorful and interesting; this dynamic nature of biological individuals that makes it hard to set clear boundaries between individuals is widely acknowledged (Dupré & O’Malley, 2009; Godfrey-Smith, 2013; Santelices, 1999; Suárez, 2020).

## Discussion

In the last section, I suggested decoupling the concept of common interest into common interest by contingency (CIC) and common interest by necessity (CIN). In both cases there is directional fitness linkage, and the demarcation line between the two is drawn by the ability of the constituent elements to withdraw from the system (at least at some stages). In this section, I want to focus on showing in what kind of relation the proposed framework stands with respect to other prominent views that are present in the literature. This will help us to understand how CIN and CIC might contribute to our growing understanding of evolution and individuality. I start by showing how the framework I propose stands in comparison to other dualistic frameworks. As mentioned before, there are many such frameworks (Godfrey-Smith, 2013; Pradeu, 2016a; Stencel & Wloch-Salamon, 2018); but I will just briefly contrast mine with that of Godfrey-Smith (2013), as it has become very influential and many who study dualistic frameworks refer in some way to this work (see for instance Okasha, 2023; Pradeu, 2016a; Smith, 2017).

Godfrey-Smith (2013) made the distinction between two types of individuals: Darwinian individuals and organisms. Darwinian individuals, as laid out in his book (Godfrey-Smith, 2009), are entities that constitute a population that undergoes evolution by natural selection. They are capable of reproduction: they causally produce entities of a similar kind. Due to this causal role in the production of offspring, they create parent–offspring lineages. Natural selection takes place at the level of Darwinian individuals: those that make more of themselves and have a higher survivability will be selected over others. A paradigm example would be a multicellular organism such as a cat. Godfrey-Smith (2013), however, believes that we can distinguish a second type of individual: organisms, which are units that are metabolically integrated. Organisms do not reproduce as a whole, but separately; and they aggregate in every generation. A paradigmatic example is a multicellular host and the majority of its symbiotic microorganisms, which are acquired from the environment yet contribute to the metabolic capabilities of the host (e.g., Amato et al., 2021; Bordenstein & Theis, 2015). The most peculiar property of organisms, in comparison to Darwinian individuals, is that they do not create lineages at the level of wholes. A microbial cell that resides in the host’s body cannot necessarily be traced back genealogically to microbes in the host’s parents, but rather to those in any member of the host population, as they can move between hosts (Sarkar et al., 2020; Tung et al., 2015).

Based on this presentation, I think it is fair to say that the core of this framework emphasizes the fact that in nature there are at least two important types of biological individuals. The line of demarcation between them rests on how they come into existence and what type of relationship they have with their ancestors. One type of entity, the Darwinian individual, is more integrated and comes into existence through direct reproduction of parents, thus creating clear parent–offspring lineages. The second type of entity, the organism, is looser, comes into existence due to aggregation and lacks a clear parent–offspring lineage. The dualistic framework that I introduced in the previous section follows the same lines of reasoning; however, they were achieved by relying purely on evolutionary considerations. On one hand, systems that exhibit common interest by necessity (CIN) are those that are highly integrated, capable of direct production of similar entities, and generate clear parent–offspring lineages. This is because such systems are so integrated that the entities which constitute them are unable to leave the system at any stage of its existence. If they are to survive and reproduce, they have to do so together. Therefore, the formation of a precise lineage across generations is a byproduct of a highly integrated and internally coherent system of entities that are unable to leave it. On the other hand, systems that exhibit common interest by contingency (CIC) are looser: they come into existence due to different forms of aggregation and they do not produce clear parent–offspring relationships across generations. They are more or less loose aggregations of entities; they interact with each other, but the system can easily disintegrate. In fact, they do not share the type of common interest that forces parts to interact with each other at all costs and so they do not produce parent–offspring lineages.

The fact that in my framework both concepts of individuality rely on the concept of fitness is also a noticeable difference between the framework I present here and that of Godfrey-Smith (2013). He does not assumes that units which constitute an organism, the metabolic individual, have to gain some benefit from belonging, in terms of fitness. Instead, the focus is merely on the certain types of physiological interactions that sustain the functionality of the whole. In contrast, my common interest framework assumes that to consider something an individual, a certain directional fitness linkage must exist. As a result of this situation, the entities individuated by the common interest framework might not coincide with those individuated by other dualistic frameworks. For instance, a given symbiotic microbe might be considered part of a metabolic unit because it contributes to the unit’s physiology (e.g., Obrenovich & Reddy, 2022; Rosenberg et al., 2007); however, in many cases, those microbes do not necessarily benefit in terms of fitness (Garcia & Gerardo, 2014; Lowe et al., 2016; Stencel, 2022). So, in the context of the common interest framework, these microbes would not be considered any sort of individual. This, in my view, shows that if you study evolution and you want to distinguish individuals based on evolutionary principles, then adopting frameworks that mix evolutionary with non-evolutionary concepts of individuals is problematic, as the latter are not relevant for fitness, which is at the heart of the theory of evolution. As result, my framework seems to be much better suited for evolutionary considerations. This does not mean that we have to discard other frameworks: they still might be useful for interdisciplinary work, where we have to link evolutionary studies with, for instance, immunology or physiology.

The other ideas that I now want to discuss briefly in relation to CIC and CIN are agency and the debate concerning the units of selection. Both these notions are constitutive of important contemporary debates in evolutionary studies and I believe that my framework might contribute to better understanding of them. I will start with concept of agency.

Recently, the study of agency has been gaining momentum in biology and philosophy, where its role and status has been widely discussed (e.g. Fulda, 2023; Okasha, 2018; Pickering, 2023; Veit, 2021). Among the issues discussed, just how to define an agent is an important one (Meincke, 2018; Okasha, 2018; Virenque & Mossio, 2023). I will focus here on one attempt to define an agent: the concept *unity-of-purpose* (Nowak & Stencel, 2022; Okasha, 2018; Veit, 2021), specifically as defined by Okasha (2018). This is because, as Okasha argued, the idea seems to be connected to the concept of common interest. According to Okasha (2018), it is justified to treat a biological unit as an agent if its various traits contribute to a single overarching goal: increasing the fitness of the organism. In other words, a biological entity is an agent if its parts perform many functions such as producing gametes or finding sexual partners or food, which are all activities that lead to increasing organism fitness. The question is; How is this status achieved if we often find many parts in organisms that promote their own fitness and which rather have a negative impact on the fitness of organism, such as selfish genes or cancer cells (Aktipis et al., 2015; Burt & Trivers, 2008; Dawkins, 1976)? We find that usually it is achieved because organisms possess mechanisms that suppress such actions, limiting the capabilities of selfish elements to spread and basically aligning their success with the success of the whole organism. This suggests that creating common interest among parts seems to be an important requirement to treat something as an agent, as stated by Okasha (2018, p. 32): “Therefore, the foregoing characterization of how suppression mechanisms work—by restricting the feasible set and thus creating a situation of common interest—can be regarded as an indirect characterization of how biological unity-of-purpose is often achieved.”

The idea that unity-of-purpose is often achieved by creating a common interest becomes interesting in the context of this paper. As I have argued throughout the paper, we should decouple that concept into two kinds of common interest: by contingency (CIC) and by necessity (CIN). So, which kind of common interest needs to be established to consider a biological unit as an agent? I think that the answer to this depends on whether we believe that agency can be lost and obtained over time by a given biological unit or whether it is something that has to persists constantly. If the latter is the case, then only biological units that exhibit CIN can be considered agents, because the fitness of their parts is directionally linked and they cannot leave the whole. Therefore, the only way they can increase their fitness is if the whole is successful; so the parts will work to enhance the fitness of the whole. If such persistence is not required, then CIC would be sufficient, as some biological units might obtain common interest temporary, like the host and the microbe. I think that assuming CIC is the kind of common interest through which agency is obtained would be problematic. Many systems can probably achieve CIC, but usually they do not have to be highly evolved and integrated. In contrast, agency in biology is usually associated with complex biological units, such as a cat (Desmond & Huneman, 2020; Okasha, 2018; Virenque & Mossio, 2023). Therefore, I think that the kind of common interest necessary for agency is CIN as biological units that exhibit it will usually consist of parts that are highly integrated and evolved to the extent that they cannot leave the system—therefore they have to work to enhance the fitness of the whole to survive. These will be agents in the way Okasha understands them.

I will now move on to the other debate: the units of selection. Many books has been written on this topic (e.g. Dawkins, 1976; Godfrey-Smith, 2009; Maynard-Smith & Szathmary, 1995; Okasha, 2006; Sober & Wilson, 1999; Suárez & Lloyd, 2023), so it would be impossible to cover even a small part of the debate here. However, I think it is justified simply to say in what sense CIN and CIC bear a relation to it and so how these concepts could possibly help provide clarification on certain issues discussed in that debate. The debate on the units of selection is about understanding which units could undergo evolution by natural selection. It is usually assumed that a population will evolve through natural selection if it consists of units that are characterized by three properties: phenotype variance, fitness differences and heritability (Lewontin, 1970; Okasha, 2006). In such a setting, as some units will do better than others, these units will pass on their phenotype traits and as a result the frequency of types will change over generations. Generally, I think that systems that exhibit either CIN or CIC could act as units of selection, if they formed part of the population characterized by the three aforementioned properties. They would constitute another level at which selection could occur; and according to multi-level selection theory there are many, from genes all the way up to ecosystems (Lloyd, 2020; Okasha, 2006; Sober & Wilson, 1999; Suárez & Lloyd, 2023; Wilson et al., 2008). However, I think CIC and CIN differ in the way they could work as a unit of selection. Generally, CIN systems always have to act as a unit of selection, due the fact that a system that exhibits CIN consists of parts that cannot leave it and join another system, or leave and exist on their own. The parts can only survive if the whole survives; therefore, either the whole is selected and reproduces or it ceases to exist. The situation with CIC is more complex and whether it acts as a unit of selection depends on the population structure. For instance, if we have a population made of systems that exhibit CIC and those systems are isolated, so their parts cannot move, we might expect that the selection will occur at the level of CIC: those that do better will contribute more to the next generation. However, if there is opportunity for movement of some parts, then systems that exhibit CIC might break down and selection might occur only at a lower level: some parts might start to increase their fitness at the expense of others (see Sect. 3 for an example of this). Therefore, systems that exhibit CIC are more susceptible to losing the status of unit of selection, due to the fact that such systems could break down, given appropriate conditions. In other words, biological units that exhibit CIN always act as a unit of selection, while those that exhibit CIC might act as a unit of selection, but only under certain circumstances.

This discussion of common interest and natural selection can help us to clarify the debate concerning the evolutionary status of holobionts—a holobiont is a combination of host and all its symbiotic microorganisms (Baedke et al., 2020). The question of whether they are units of selection has been studied very intensely, with some people arguing in favor (Brucker & Bordenstein, 2013; Rosenberg & Zilber-Rosenberg, 2018; Suárez, 2020; Triviño & Suárez, 2020) and some against (Douglas & Werren, 2016; Moran & Sloan, 2015; Skillings, 2016; Stencel & Wloch-Salamon, 2018). The interesting part of the debate is that many people referred to common interest in order to argue that holobionts cannot be units of selection (e.g. Foster et al., 2017; Skillings, 2016). For instance, Foster et al., (2017, p. 5) wrote: “ (…) we cannot assume that the host and microbiota are a single evolutionary unit acting with a common interest, as is sometimes done in applications of the ‘holobiont’ or ‘superorganism’ metaphors^23^.” In the context of this paper, we could ask what kind of common interest holobionts lack and whether a lack of it implies that they cannot be a unit of selection? One clear fact about holobionts is that the majority of the microbes are not tightly integrated into the host (Amato et al., 2021; Obrenovich & Reddy, 2022; Sarkar et al., 2020), but rather they can move from one host to another—a small exception would be highly integrated, vertically inherited symbionts like *Buchnera sp.* in aphids (Bennett & Moran, 2015; Chong et al., 2019). This suggests that holobionts definitely do not exhibit CIN, which is specifically the case why some people rejected the idea that the holobiont could be a unit of selection. For example, Skillings (p. 9) wrote: “If partner lineages can jump ship, and horizontally transfer to other hosts, then the different parts of a holobiont aren’t locked into a common fate. This leads to an expectation of increased conflict between the members of the holobiont as they “pursue their own goals” (…) As conflicts of interests among partners increase (e.g., due to weak partner fidelity), then the holobiont is undermined as a higher-level unit of selection.” There are two points that could be made here to defend the idea that a holobiont could be a unit of selection, at least in some circumstances. Firstly, as I argue above, common interest is not equal to the concept of unit of selection: selection could act beyond or below the biological units that share a common interest. Secondly, even if someone argues that we should keep the idea that common interest is necessary to consider something as a unit of selection, we have two kinds of common interest: CIC and CIN. While holobionts are unlikely to exhibit CIN, the host and the microbes might often form systems that exhibit CIC (see Sect. 3 for an example). A conclusion would therefore be that the holobiont is not *always* a unit of selection, but could be sometimes, given the appropriate population conditions, as that is how systems that exhibit CIC act as a unit of selection (as I argue above); for example, if the population conditions provide variance at the level of CIC and minimize the tendency of such systems to break down. This conclusion corresponds with many theoretical models that assume that holobionts can act as units of selection under certain conditions (e.g. Huitzil et al., 2018; Lloyd & Wade, 2019; Roughgarden et al., 2018).

At the end of this section, I would like to return to the discussion of the concept of common interest itself. In particular, I aim to highlight both the conceptual advances introduced by my work and its limitations. Let us begin with the former.

The concept of common interest is widely used in the literature, especially in discussions of evolutionary individuality (Bourrat & Griffiths, 2018; Foster et al., 2017; Schenkel et al., 2025; Skillings, 2016; Stencel & Wloch-Salamon, 2018). Therefore, it is important to make clear how this paper advances the current state of the debate. I believe the paper’s main contribution lies in showing that the notion of common interest is far more nuanced than it is typically assumed to be. Entities that share a common interest can differ substantially in their nature. For this reason, I emphasize the need to distinguish between two types of common interest: common interest by necessity and common interest by contingency. This distinction represents a departure from previous work, as no one has yet argued for introducing two distinct forms of common interest. This lack of distinction is not surprising. Although the concept of common interest has been widely applied, its underlying nature has rarely been examined in detail. This sets it apart from other concepts, such as altruism, which have received much greater analytical attention (Kerr et al., 2004; West et al., 2006). The more fine-grained framework I propose should therefore be of significant value to researchers employing the concept of common interest, as it offers a novel way of understanding it. Regardless of whether one accepts my further conclusions—for instance, that entities sharing a common interest should be considered evolutionary individuals distinct from agents or units of selection (as discussed above)—one should at least acknowledge my attempt to deepen the theoretical study of the concept of common interest.

The framework I developed in this paper also has some limitations. One of them, which I would like to discuss here, concerns how to conceptualize the notion of common interest. In this paper, I focused on the alignment of inclusive fitness as a measure of common interest. However, using inclusive fitness is not the only possible approach. For instance, one could measure common interest by focusing on other metrics, such as persistence (e.g. Bouchard, 2014; Doolittle & Booth, 2017; Jorge et al., 2020). Alternatively, one could employ more specific frameworks, such as the one developed by Queller and Strassmann. They argued that common goals emerge when individuals exhibit a high level of cooperation and a low level of conflict (Queller & Strassmann, 2009, 2016). If we change the way we conceptualize common interest, it is very likely that we might reach different conclusions. Therefore, it will be necessary to evaluate in the future whether the approach I have taken is indeed the most appropriate. At present, I believe it is, since it is grounded in the well-established concept of inclusive fitness, which appears to be well suited for analyzing evolutionary issues (see Abbot et al., 2011; Gardner & West, 2014; West & Gardner, 2013).

## Concluding remarks

The last piece of the puzzle in this paper is to show how the framework I have developed fits into the general landscape of the debate on biological individuality. Pradeu (2016a, p. 797) stated that the question about biological individuality is ultimately about asking: “What, in the living world, constitutes a relatively well-delineated and cohesive unit?”. There is a growing discussion about individuality in different fields, such as immunology (Pradeu, 2010; Swiatczak & Tauber, 2020; Veigl, 2022), and a consensus is that what constitutes a “cohesive unit” will be different for different fields and that many concepts of organism could co-exist because they fulfil different epistemological goals (Kovaka, 2015; Pepper & Herron, 2008; Pradeu, 2016b; Stencel & Proszewska, 2018). Probably there is no single concept that could satisfy every research need. In this paper I have tried to develop evolutionary dualism. My framework is aimed at evolutionary studies, and says nothing about the status of the concepts of individuality in different fields—my work could be understood as an enrichment of the pluralistic paradigm. In other words, while I defended a view of individuality based on common interests that outline specific conditions for considering something an individual, I think there might be many more types of non-evolutionary individuals that could be individuated according to different criteria (such as those from immunology, developmental biology, etc.).

In evolutionary biology, people often associate evolutionary individuality with the unit of selection (e.g. Bourrat & Griffiths, 2018; Ereshefsky & Pedroso, 2015; Godfrey-Smith, 2009). In this paper, I have done something a bit different, as can be seen in the last section. I have argued that evolutionary individuality could be related both to agency and to units of selection; however, it is not exactly the same concept in the two cases. Agency is about purposefulness and teleological systems in biology (Fulda, 2023; Okasha, 2018; Veit, 2021), whereas the debate on the units of selection is about figuring out which populations can undergo evolution by natural selection (Godfrey-Smith, 2009; Lewontin, 1970; Okasha, 2018). Both these are important issues that are widely studied. However, among the discussions, people often come up with the idea that the evolutionary future and fate of some entities depends reciprocally on those of others, and this makes them evolutionarily important units (e.g. Dawkins, 1990; Foster et al., 2017; Patten et al., 2023; Skillings, 2016; Stencel & Wloch-Salamon, 2018). This is where the concept of common interest is brought in: to emphasis this aspect. Sometimes, this idea is linked to the debate concerning units of selection (e.g. Foster et al., 2017; Skillings, 2016; Stencel & Wloch-Salamon, 2018); sometimes it is discussed in the context of evolutionary considerations but without any link to the debate on units of selection (e.g. Anderson, 2006; Van Baalen & Jansen, 2001). This shows that units that exhibit common interest have some kind of evolutionary “cohesion” that pushes scientists toward individuating them. Therefore, I think they deserve to be treated as meaningful units and that is how I treat them in this paper. Evolutionary individuals are units characterized by common interest, which may or may not also be units of selection and/or agents (I agree with those who think there are few evolutionarily important units; see Lloyd, 2020; Suárez & Lloyd, 2023). I have argued that there are two kinds of common interest that corresponds to two kinds of evolutionary individuals. Systems that exhibit CIN are units which, due to their evolutionary history, have an inseparable fate; systems that exhibit CIC share an evolutionary future only temporary, and if different conditions occur, that future might break down. Of course, on an evolutionary scale, everything might change, as I have discussed; but at least at a certain point of evolutionary history we should be able to spot these two kinds of evolutionary individuals.

## Footnotes

1 This means that, in my approach, individuals are systems. It does not imply that every system is an evolutionary individual; to be so considered it has to fulfil the condition I will set out below.

2 See the next section for an example of this.
